# A survey of perceptions and attitudes about direct-to-consumer advertising of prescription drugs among college students in South Korea

**DOI:** 10.1371/journal.pone.0201108

**Published:** 2018-07-24

**Authors:** Young-Mo Yang, Jae-Joon Lee, Eun Jeong, Sun Young Kim, Mi Ah Han, Eun Joo Choi

**Affiliations:** 1 Department of Pharmacy, College of Pharmacy, Chosun University, Gwangju, South Korea; 2 Department of Food and Nutrition, Chosun University, Gwangju, South Korea; 3 Department of Biology Education, Chosun University, Gwangju, South Korea; 4 Department of Preventive Medicine, College of Medicine, Chosun University, Gwangju, South Korea; Universitat Luzern, SWITZERLAND

## Abstract

Direct-to-consumer advertising (DTCA) of prescription drugs can be both beneficial and harmful to healthcare consumers. Therefore, DTCA for prescription drugs is a topic that should be considered crucially, at this point, when the interests of patients as well as pharmaceutical companies in DTCA of prescription drugs are growing in South Korea. The goals of this study were to investigate Korean college students’ perceptions and attitudes about DTCA of prescription drugs through a survey as well as to analyze data according to their college majors in order to identify differences in their perceptions and attitudes about prescription drug DTCAs as future health care professionals and consumers, respectively. A descriptive, cross-sectional survey was conducted between September and November 2015. Participants were recruited from Chosun University in Gwangju, South Korea. Ethical approval for this study was obtained from the Chosun University Institutional Review Board. Of 1,040 questionnaires initially distributed, 774 were collected, and 742 were included in the analysis. The results of this study indicated that most students who had participated in the survey did not have sufficient knowledge of DTCA for prescription drugs. Approximately, 17% reported being cognizant of DTCA for prescription drugs. More healthcare students (24.6%) knew this term than non-healthcare students did (6.3%). In this study, most of the students were likely to feel that healthcare professionals (e.g., doctors and pharmacists) had the responsibility of delivering information about prescription drugs to patients, and that all prescription drugs DTCA, if it were permitted, had to be pre-approved by the Korean government. The results of this study indicated that DTCA for prescription drugs had to be permitted under the condition of pre-approval of the DTCA contents by the Korean government, and prescription drugs should not be advertised through the Internet. It is recommended that the Korean government cautiously examine whether DTCA of prescription drugs should be permitted, after considering the current marketing strategies of pharmaceutical companies on the Internet and the effects of online electronic-DTCA on Korean consumers.

## Introduction

In the United States (U.S.), drug advertising to physicians and patients is a popular marketing strategy in the pharmaceutical industry, which spends approximately twice as much on advertising than on research and development [1,2]. Direct-to-consumer advertising (DTCA) for prescription drugs can be defined as prescription drugs advertising targeted directly at consumers through public media, such as television, radio, newspaper, and Internet [3]. Pharmaceutical companies use rational (e.g., statements on the benefits of taking their products) and emotional (e.g., combined visual imagery, music, and spoken words) appeals in order to persuade consumers to choose their products; consequently, these advertisements may provide misleading information, increase the prescription frequency of advertised drugs, and have a significant effect on requests for newer and more expensive drugs that are not necessarily more effective than conventional ones [4–7].

DTCA of prescription drugs is currently permitted only in the U.S. and New Zealand, but various promotional techniques (e.g., promotional materials, reminder advertisements, and unbranded advertising campaigns) can be employed in other countries [3,5,6]. The European Union had considered adopting it for limited prescription drugs associated with AIDS, diabetes, and asthma; however, its members emphatically refused to accept this idea in order to prevent potential harm to the public due to exposure to the DTCA of prescription drugs [8–10]. The Korean government has kept a slightly conservative stance on the approval of DTCA of prescription drugs since 1990 [9]. In Korea, prescription drugs can be advertised only in professional medical and healthcare journals, with the exception of drugs prescribed for the prevention of contagious diseases (e.g., cholera, plague, typhoid fever, influenza, and AIDS), which are also allowed to be directly advertised for consumers [9].

Since the Korean government implemented the Korean Health Care System Reform Act of 2000, better known as “Separation of Prescribing and Dispensing”, patients have been able to access their own prescriptions freely [11]. Additionally, they can obtain information about their prescribed drugs through the Internet, even though DTCA of prescription drugs is only legally permitted in the two countries mentioned above. Moreover, due to rapidly developing social networking sites (e.g., Facebook, Twitter, and YouTube) and interactive systems, it is expected that the Internet will soon remove information barriers to the DTCA for prescription drugs [9,12,13]. According to a previous study conducted with individuals living in Sacramento, California (U.S.) and Vancouver, British Columbia (Canada) [14], Koreans exposed to more DTCA of prescription drugs through the Internet are likely to request more of the advertised medications. Therefore, DTCA of prescription drugs is a crucial topic that should be considered, at this point, given the increasing interest of patients as well as pharmaceutical companies in the DTCA of prescription drugs.

Several studies on perceptions and attitudes about DTCA of prescription drugs have been conducted in various countries [15–23]. In Korea, however, relevant studies have been rarely conducted [9,24,25]. Thus, the present study investigated Korean college students’ perceptions and attitudes about DTCA of prescription drugs through a survey, and analyzed the data according to the students’ college majors (healthcare and non-healthcare) in order to attempt to identify differences in their perceptions and attitudes about prescription drug DTCA as future healthcare professionals and consumers, respectively.

## Materials and methods

### Study population and recruitment

A descriptive, cross-sectional survey was carried out between September and November 2015 in order to fulfill the objectives of this study. Participants were recruited from Chosun University in Gwangju, South Korea. Eligible participants were college students who had agreed to participate in the study and could commute to college in order to attend lectures. Healthcare and non-healthcare students were recruited in healthcare and non-healthcare lectures, respectively. No statistical sampling procedures were performed. Ethical approval for this study was received from the Chosun University Institutional Review Board (2-1041055-AB-N-01-2015-0008).

### Study tool development and data collection

A survey questionnaire was developed based on previously published research [9,19,23,25,26]. A pilot test of the questionnaire was implemented with ten students, who were not part of the study sample, in order to verify content validity and clarity. Minor revisions were made based upon the pilot test results. The questionnaire collected demographic data (sex, age, college, and academic year), health status, knowledge about DTCA for prescription drugs, and also included questions regarding the students’ perceptions of and attitudes toward DTCA for prescription drugs. Responses to the questions were scored using 5-point Likert scales (5 = Strongly agree, 4 = Agree, 3 = Neutral, 2 = Disagree, 1 = Strongly disagree), except for demographic data and knowledge about DTCA. The self-report questionnaires were distributed to the students together with consent forms once the concept of DTCA for prescription drugs as well as the objectives of the study had been verbally explained for a sufficient time before the beginning of the lecture. Those who did not agree to participate in the survey were excluded. The survey was completed anonymously and voluntarily, and it took approximately five minutes to answer all questions. An attendance count was carried out to estimate response rates.

### Statistical analysis

Demographic data and responses were coded to prevent the identification of participants, and all analyses were carried out using SPSS for Windows, version 20.0 (SPSS, Inc., Chicago). Categorical variables were summarized using frequencies (n) and percentages (%), whereas continuous variables were described using means (SD). The Chi-square test and student’s *t*-test or Welch’s *t*-test were employed to assess differences in proportions and means, respectively, between the healthcare and the non-healthcare groups. The student’s *t*-test or Welch’s *t*-test was also utilized to compare differences in means between freshman/sophomore and junior/senior groups. The student’s *t*-test was used to account for equal variances, and the Welch’s *t*-test was utilized to explain unequal variances. The healthcare group included pharmacy, medical, and dental students, and the non-healthcare group included all students other than those who were in the healthcare group. Additionally, an analysis of covariance (ANCOVA) was performed to assess whether there were significant differences in the mean responses to the questions regarding perceptions and attitudes, adjusting for sex, age, and knowledge about DTCA for prescription drugs. Any missing data were not estimated or included in the analysis. Statistical significance was assumed to be *p* < 0.05.

## Results

Of 1,040 questionnaires initially distributed, 774 were collected, and 742 valid surveys were included in the analysis. The respondents’ characteristics are shown in Table 1. Of the 742 respondents, 419 belonged to the healthcare group, and 323 were in the non-healthcare group. Mean ages of the healthcare and non-healthcare groups were 25.51 (3.75) and 20.25 (2.91) years old, respectively. Approximately, 56% were women, and about 49% were freshmen. Only about 17% were familiar with DTCA for prescription drugs, and most of those were healthcare students.

**Table 1:** Descriptive statistics of the survey respondents’ characteristics.

Characteristics	Overall	Healthcare	Non-healthcare	*p*-value
Age				
Median (range), years	23 (17–59)	25 (18–40)	19.5 (17–59)	
Mean (SD), years	23.21 (4.29)	25.51 (3.75)	20.25 (2.91)	< 0.001^*^
10 s, n (%)	183/734 (24.9)	21/411 (5.1)	162/323 (50.2)	< 0.001
20 s, n (%)	489/734 (66.6)	330/411 (80.3)	159/323 (49.2)	
30 s or above, n (%)	62/734 (8.4)	60/411 (14.6)	2/323 (0.6)	
Sex, n (%)				
Men	328/742 (44.2)	227/419 (54.2)	101/323 (31.3)	< 0.001
Women	414/742 (55.8)	192/419 (45.8)	222/323 (68.7)	
Academic year, n (%)				
Freshman	362/741 (48.9)	196/418 (46.9)	166/323 (51.4)	< 0.001
Sophomore	128/741 (17.3)	71/418 (17.0)	57/323 (17.6)	
Junior	121/741 (16.3)	89/418 (21.3)	32/323 (9.9)	
Senior	130/741 (17.5)	62/418 (14.8)	68/323 (21.1)	
Health status, n (%)				
Poor	24/740 (3.2)	17/417 (4.1)	7/323 (2.2)	0.372
Acceptable	209/740 (28.2)	114/417 (27.3)	95/323 (29.4)	
Good	303/740 (40.9)	166/417 (39.8)	137/323 (42.4)	
Very good	204/740 (27.6)	120/417 (28.8)	84/323 (26.0)	
Have you heard about direct-to-consumer advertising (DTCA) for prescription drugs?, n (%)				
Yes	123/737 (16.7)	103/418 (24.6)	20/319 (6.3)	< 0.001
No	614/737 (83.3)	315/418 (75.4)	299/319 (93.7)	

SD, standard deviation; DTCA, direct-to-consumer advertising.

^*^t (731.938) = 21.168.

The results related to the participants’ perceptions regarding prescription drug DTCA are presented in Table 2. Most of the respondents felt that doctors or pharmacists had to give patients information about prescription drugs (mean score, 4.30), and there was no significant difference between healthcare and non-healthcare students. However, the non-healthcare students were more likely than the healthcare students to agree with the following questions (mean score healthcare vs. mean score non-healthcare): Q2 (3.49 vs. 3.68), Q3 (3.07 vs. 3.67), Q4 (3.44 vs. 3.77), Q7 (3.16 vs. 3.65), Q8 (3.26 vs. 3.72), and Q10 (2.78 vs. 3.19), and the comparison between the two groups found significant differences in the mean responses on these six questions. Conversely, the healthcare students were more likely than the non-healthcare students to agree with the following questions (mean score healthcare vs. mean score non-healthcare): Q5 (3.30 vs. 2.87), Q9 (3.82 vs. 3.64), and Q11 (3.38 vs. 3.12), and there were also significant differences in the mean responses between both groups on these three questions. The largest difference between the mean responses of the two groups was found for question Q3 (mean difference, 0.60). In other words, the non-healthcare students were more likely than the healthcare students to agree that prescription drug DTCA could encourage patients to follow the treatment instructions or advice of their doctors. The healthcare students showed the most skeptical perception of the idea that prescription drug DTCA would be able to play a role in removing the rebates of drug companies to doctors (mean score, 2.78), and the non-healthcare students showed the most doubtful perception of the idea that prescription drug DTCA could disrupt the doctor-patient relationship (mean score, 2.87). The results related to the perceptions regarding DTCA for prescription drugs between the freshman/sophomore and junior/senior groups are also summarized in Table 2.

**Table 2:** Perceptions of Korean college students about DTCA for prescription drugs.

Question	Overall, Mean (SD) (n = 742)	Healthcare, Mean (SD) (n = 419)	Non-healthcare, Mean (SD) (n = 323)	*t*	*df*	*p*-value	Freshman and Sophomore						Junior and Senior					
							Overall, Mean (SD) (n = 490)	Healthcare, Mean (SD) (n = 267)	Non-healthcare, Mean (SD) (n = 223)	*t*	*df*	*p*-value	Overall, Mean (SD) (n = 251)	Healthcare, Mean (SD) (n = 151)	Non-healthcare, Mean (SD) (n = 100)	*t*	*df*	*p*-value
Q1. Do you think that doctors or pharmacists have to deliver information about prescription drugs to patients?	4.30 (0.84)	4.30 (0.84)	4.31 (0.84)	-0.269	740	0.788	4.32 (0.83)	4.32 (0.83)	4.31 (0.84)	0.108	488	0.914	4.27 (0.85)	4.25 (0.85)	4.31 (0.86)	-0.590	249	0.555
Q2. Do you think that DTCA for prescription drugs can give patients confidence to talk with doctors about their concerns?	3.57 (0.89)	3.49 (0.95)	3.68 (0.79)	-3.031	735.618	0.003	3.60 (0.88)	3.53 (0.94)	3.68 (0.79)	-1.914	487.996	0.056	3.51 (0.91)	3.40 (0.96)	3.68 (0.80)	-2.435	249	0.016
Q3. Do you think that DTCA for prescription drugs can encourage patients to follow treatment instructions or advice from their doctors?	3.33 (1.01)	3.07 (1.05)	3.67 (0.84)	-8.623	739.297	< 0.001	3.38 (0.99)	3.10 (1.04)	3.71 (0.81)	-7.327	485.366	< 0.001	3.23 (1.04)	3.00 (1.07)	3.57 (0.90)	-4.390	249	< 0.001
Q4. Do you think that DTCA for prescription drugs can improve patients’ awareness of medical conditions?	3.59 (0.91)	3.44 (0.95)	3.77 (0.82)	-5.071	730.610	< 0.001	3.63 (0.88)	3.48 (0.93)	3.82 (0.79)	-4.460	487.902	< 0.001	3.49 (0.96)	3.38 (0.99)	3.67 (0.89)	-2.394	249	0.017
Q5. Do you think that DTCA for prescription drugs can interfere with the relationships between patients and doctors?	3.11 (1.03)	3.30 (1.07)	2.87 (0.93)	5.800	728.704	< 0.001	3.09 (1.04)	3.23 (1.09)	2.92 (0.95)	3.308	486.732	0.001	3.16 (1.02)	3.42 (1.02)	2.76 (0.87)	5.527	234.371	< 0.001
Q6. Do you think that DTCA for prescription drugs can promote unnecessary visits to hospitals?	3.16 (0.95)	3.18 (0.96)	3.14 (0.93)	0.680	740	0.497	3.18 (0.94)	3.16 (0.96)	3.20 (0.92)	-0.433	488	0.665	3.13 (0.96)	3.22 (0.96)	2.99 (0.94)	1.866	249	0.063
Q7. Do you think that DTCA for prescription drugs can prevent incorrect information on drugs from being spread?	3.38 (1.03)	3.16 (1.08)	3.65 (0.89)	-6.784	737.367	< 0.001	3.46 (1.01)	3.27 (1.06)	3.68 (0.90)	-4.650	487.827	< 0.001	3.21 (1.05)	2.96 (1.10)	3.59 (0.85)	-5.103	242.322	< 0.001
Q8. Do you think that DTCA for prescription drugs can broaden patients’ choices for using drugs?	3.46 (0.97)	3.26 (1.03)	3.72 (0.81)	-6.813	739.715	< 0.001	3.49 (0.95)	3.28 (1.02)	3.74 (0.80)	-5.541	486.544	< 0.001	3.39 (1.00)	3.20 (1.05)	3.67 (0.83)	-3.954	241.449	< 0.001
Q9. Do you expect that DTCA for prescription drugs will increase the profits of pharmaceutical companies?	3.74 (0.86)	3.82 (0.89)	3.64 (0.82)	2.877	740	0.004	3.70 (0.85)	3.76 (0.88)	3.63 (0.82)	1.606	488	0.109	3.83 (0.87)	3.94 (0.90)	3.66 (0.81)	2.524	249	0.012
Q10. Do you think that DTCA for prescription drugs can play a role in removing the rebates of pharmaceutical companies to doctors?	2.96 (0.99)	2.78 (1.05)	3.19 (0.85)	-5.917	738.092	< 0.001	2.98 (0.99)	2.73 (1.03)	3.26 (0.85)	-6.243	487.841	< 0.001	2.91 (0.99)	2.83 (1.07)	3.02 (0.84)	-1.535	241.526	0.126
Q11. Do you think that DTCA for prescription drugs can have a negative effect on patients’ drug misuse/abuse?	3.27 (1.00)	3.38 (1.00)	3.12 (0.98)	3.555	699.838	< 0.001	3.27 (1.03)	3.34 (1.04)	3.17 (1.01)	1.875	488	0.061	3.28 (0.95)	3.46 (0.94)	3.02 (0.91)	3.682	216.309	< 0.001

Mean scores were based on the following scale: 5 = Strongly agree, 4 = Agree, 3 = Neutral, 2 = Disagree, 1 = Strongly disagree; DTCA, direct-to-consumer advertising; SD, standard deviation; ***df***, degrees of freedom.

The results regarding the participants’ attitudes toward DTCA for prescription drugs are depicted in Table 3. Most of the respondents tended to feel that the government had to mandate pre-approval of all prescription drug DTCA if it were to be permitted (mean score, 3.97), and there was a significant difference between the healthcare and non-healthcare students. The mean responses to all questions regarding attitudes toward prescription drug DTCA revealed significant differences. The non-healthcare students were more likely than the healthcare students to agree with the following questions (mean score healthcare vs. mean score non-healthcare): Q12 (3.22 vs. 3.72), Q13 (3.05 vs. 3.43), Q19 (3.25 vs. 3.39), and Q20 (2.74 vs. 3.23). However, the healthcare students were more likely than the non-healthcare students to agree with the following questions (mean score healthcare vs. mean score non-healthcare): Q17 (3.46 vs. 3.12), Q18 (3.63 vs. 3.27), and Q21 (4.08 vs. 3.83). The biggest differences in the mean responses between the two groups were found for questions Q12 (mean difference, 0.50) and Q20 (mean difference, 0.49). The non-healthcare students were more likely than the healthcare students to agree with the attitudes toward the need of patients for prescription drug DTCA and toward the lowering of drug prices due to increased market competition caused by DTCA for prescription drugs. The healthcare students showed the most doubtful attitude toward the lowering of drug prices (mean score, 2.74). In addition, the results with relation to attitudes toward DTCA for prescription drugs between the freshman/sophomore and junior/senior groups are presented in Table 3.

**Table 3:** Attitudes of Korean college students toward DTCA for prescription drugs.

Question	Overall, Mean (SD) (n = 742)	Healthcare, Mean (SD) (n = 419)	Non-healthcare, Mean (SD) (n = 323)	*t*	*df*	*p*-value	Freshman and Sophomore						Junior and Senior					
							Overall, Mean (SD) (n = 490)	Healthcare, Mean (SD) (n = 267)	Non-healthcare, Mean (SD) (n = 223)	*t*	*df*	*p*-value	Overall, Mean (SD) (n = 251)	Healthcare, Mean (SD) (n = 151)	Non-healthcare, Mean (SD) (n = 100)	*t*	*df*	*p*-value
Q12. Do you think that DTCA for prescription drugs is necessary for patients?	3.44 (0.92)	3.22 (0.98)	3.72 (0.76)	-7.786	739.895	< 0.001	3.48 (0.93)	3.24 (0.98)	3.78 (0.77)	-6.823	486.624	< 0.001	3.35 (0.90)	3.19 (0.97)	3.60 (0.73)	-3.590	249	< 0.001
Q13. Do you think that information about drugs provided by DTCA is reliable?	3.22 (0.92)	3.05 (0.97)	3.43 (0.80)	-5.612	740	< 0.001	3.27 (0.95)	3.06 (1.02)	3.51 (0.81)	-5.377	486.547	< 0.001	3.12 (0.85)	3.03 (0.89)	3.25 (0.77)	-2.058	249	0.041
Q14. Are you willing to actively recommend DTCA for prescription drugs when patients ask you about it in the future?	-	3.03 (1.02)	-			-	-	2.99 (1.03)	-			-	-	3.08 (1.00)	-			-
Q15. Are you willing to actively utilize the data obtained from DTCA for prescription drugs when consulting patients in the future?	-	3.30 (0.92)	-			-	-	3.30 (0.95)	-			-	-	3.30 (0.85)	-			-
Q16. Are you willing to actively accept patients’ opinions when they ask you to prescribe, fill, or administer drugs which they have seen on DTCA in the future?	-	3.19 (0.87)	-			-	-	3.10 (0.88)	-			-	-	3.34 (0.81)	-			-
Q17. Do you think that DTCA for prescription drugs should not be permitted on the Internet?	3.31 (1.04)	3.46 (1.08)	3.12 (0.95)	4.563	727.635	< 0.001	3.25 (1.04)	3.38 (1.09)	3.10 (0.96)	3.064	486.625	0.002	3.42 (1.03)	3.60 (1.06)	3.16 (0.93)	3.440	230.245	0.001
Q18. Do you think that DTCA for prescription drugs can create unrealistic expectations about drugs?	3.47 (0.94)	3.63 (0.99)	3.27 (0.84)	5.404	732.676	< 0.001	3.45 (0.97)	3.59 (1.04)	3.28 (0.85)	3.625	487.664	< 0.001	3.52 (0.89)	3.71 (0.88)	3.24 (0.83)	4.212	249	< 0.001
Q19. Do you think that DTCA for prescription drugs can improve patients’ drug compliance?	3.31 (0.87)	3.25 (0.94)	3.39 (0.76)	-2.266	738.414	0.024	3.34 (0.89)	3.22 (0.97)	3.49 (0.75)	-3.394	484.528	0.001	3.24 (0.84)	3.28 (0.89)	3.17 (0.75)	1.099	234.461	0.273
Q20. Do you expect that DTCA for prescription drugs will lead to lowering drug prices due to increased market competition?	2.95 (1.02)	2.74 (1.05)	3.23 (0.91)	-6.857	729.633	< 0.001	2.96 (1.02)	2.70 (1.04)	3.27 (0.90)	-6.590	487.551	< 0.001	2.94 (1.03)	2.80 (1.07)	3.14 (0.94)	-2.580	249	0.010
Q21. Do you think that the government should mandate pre-approval of all DTCAs for prescription drugs if they are permitted?	3.97 (0.93)	4.08 (0.95)	3.83 (0.88)	3.663	740	< 0.001	4.00 (0.90)	4.13 (0.88)	3.84 (0.90)	3.675	488	< 0.001	3.91 (0.97)	3.97 (1.06)	3.81 (0.83)	1.371	242.342	0.172

Mean scores were based on the following scale: 5 = Strongly agree, 4 = Agree, 3 = Neutral, 2 = Disagree, 1 = Strongly disagree; DTCA, direct-to-consumer advertising; SD, standard deviation; ***df***, degrees of freedom.

In order to determine whether significant differences in the mean responses to the questions concerning perceptions and attitudes between healthcare and non-healthcare students existed, an ANCOVA was run after adjusting for sex, age, and knowledge about prescription drug DTCA, and the results are shown in Table 4. The adjusted means for all questions except for questions Q1 and Q6 were significantly different between the two groups.

**Table 4:** Comparison of Korean college students’ perceptions and attitudes about DTCA for prescription drugs.

Question	Overall, Mean (SE)	Healthcare, Mean (SE)	Non-healthcare, Mean (SE)	*F*	*p*-value	Partial Eta Squared
Q1	4.30 (0.03)	4.30 (0.04)	4.30 (0.05)	0.002	0.967	< 0.001
Q2	3.58 (0.03)	3.50 (0.04)	3.66 (0.05)	5.823	0.016	0.008
Q3	3.36 (0.04)	3.10 (0.05)	3.63 (0.06)	50.443	< 0.001	0.065
Q4	3.61 (0.03)	3.46 (0.05)	3.76 (0.05)	18.000	< 0.001	0.024
Q5	3.09 (0.04)	3.27 (0.05)	2.92 (0.06)	20.532	< 0.001	0.027
Q6	3.17 (0.04)	3.16 (0.05)	3.18 (0.05)	0.078	0.780	< 0.001
Q7	3.40 (0.04)	3.18 (0.05)	3.62 (0.06)	32.045	< 0.001	0.042
Q8	3.49 (0.04)	3.26 (0.05)	3.71 (0.05)	37.411	< 0.001	0.049
Q9	3.73 (0.03)	3.81 (0.04)	3.66 (0.05)	5.219	0.023	0.007
Q10	2.98 (0.04)	2.78 (0.05)	3.19 (0.06)	29.095	< 0.001	0.038
Q11	3.26 (0.04)	3.36 (0.05)	3.17 (0.06)	5.928	0.015	0.008
Q12	3.47 (0.03)	3.24 (0.05)	3.71 (0.05)	45.568	< 0.001	0.059
Q13	3.24 (0.03)	3.06 (0.05)	3.42 (0.05)	25.775	< 0.001	0.034
Q17	3.30 (0.04)	3.43 (0.05)	3.16 (0.06)	11.293	0.001	0.015
Q18	3.46 (0.03)	3.59 (0.05)	3.33 (0.05)	13.276	< 0.001	0.018
Q19	3.32 (0.03)	3.24 (0.04)	3.40 (0.05)	5.533	0.019	0.008
Q20	2.98 (0.04)	2.76 (0.05)	3.20 (0.06)	33.150	< 0.001	0.043
Q21	3.96 (0.03)	4.07 (0.05)	3.85 (0.05)	9.575	0.002	0.013

An ANCOVA was performed and the table shows mean (SE) adjusted to sex, age, and knowledge on DTCA for prescription drugs; DTCA, direct-to-consumer advertising; SE, standard error.

## Discussion

In this study, Korean college students’ perceptions and attitudes about prescription drug DTCA were examined through a survey and the data was analyzed in terms of their college majors (healthcare vs. non-healthcare) in order to determine whether disparities existed between these two groups, which represent future healthcare professionals and consumers, respectively. Although similar studies were previously conducted with Korean healthcare providers (i.e., pharmacists and doctors) and patients [9,24], it is believed that this is the first study to investigate Korean college students’ perceptions and attitudes about prescription drug DTCA. Due to the results of previous studies and since the Internet can rapidly remove informational barriers to prescription drugs, the results presented here may help to further delineate both the positive and negative effects of DTCA for prescription drugs in countries, such as Korea, where this practice is currently not allowed.

The results of this study indicated that most students who participated in the survey did not have sufficient knowledge about DTCA for prescription drugs. Approximately, 17% reported that they were familiar with DTCA for prescription drugs. More healthcare students (24.6%) tended to know this term than non-healthcare students (6.3%). Most of the students in this study were likely to feel that healthcare professionals (e.g., doctors or pharmacists) had the responsibility to deliver information about prescription drugs to patients, and that all prescription drug DTCA had to be pre-approved by the Korean government if it were to be permitted.

The findings of this study suggested that when compared with non-healthcare students (mean score, 3.67), healthcare students (mean score, 3.07) had a more negative perception of the idea that DTCA of prescription drugs could encourage patients to follow the treatment instructions or advice of their doctors. This tendency can be explained to some degree by the fact that healthcare students had more negative thoughts on patients’ drug misuse/abuse, the improvement of patients’ awareness of medical conditions, and the prevention of spreading incorrect information about drugs through DTCA. The disadvantages of allowing DTCA for prescription drugs may include a higher risk for drug misuse and abuse, the patients’ acceptance of limited information about drugs without considering healthcare professionals’ knowledge, and the invasion of doctors’ rights to prescribe medications [9]. In particular, emotional appeals used in DTCA may lead consumers to ignore crucial information regarding risks and benefits that need to be considered in order to select appropriate drugs, which may possibly lead consumers to prefer advertised products [5,27]. Consequently, this is likely to induce consumers, including those unlikely to be at risk of the condition, to seek medical attention for clinically inappropriate reasons, such as fear derived from not using the advertised products [5].

An interesting finding in this study was that the majority of students in both the healthcare and non-healthcare groups felt highly skeptical regarding the idea that DTCA for prescription drugs could play a role in eliminating the rebates of pharmaceutical companies to doctors (mean score, 2.96). Specifically, healthcare students believed that DTCA would be more unlikely to lead to prohibiting drug companies from providing compensation to physicians who prescribed specific drugs. This result aligned with that of a previous study conducted with Korean patients who had visited community pharmacies to fill prescriptions [9]. Another intriguing finding in this study was that compared to healthcare students, non-healthcare students felt that DTCA for prescription drugs would be more unlikely to interfere with the patient-doctor relationship. While it is difficult to directly compare this result with that from a study conducted with Korean healthcare providers and consumers due to the different survey subjects, a comparison might be possible given that healthcare and non-healthcare students are likely to be future healthcare providers and consumers, respectively; furthermore, in this case, the results from the present study were different from those of the previous study. In fact, healthcare providers (e.g., pharmacists and doctors) tended to feel that DTCA would be more unlikely to disrupt the relationship between doctors and patients [24]. The reason for this difference cannot be currently examined since DTCA is not allowed in Korea. Thus, this point will have to be addressed in future studies if and when DTCA is allowed in Korea.

In terms of the Korean college students’ attitudes toward DTCA for prescription drugs, the majority of both healthcare and non-healthcare students felt that pre-approval of all DTCA for prescription drugs had to be carried out by the Korean government if DTCA were to be allowed (mean score, 3.97). Further, compared with healthcare students, non-healthcare students showed a slightly more negative attitude toward this question. A similar tendency was shown in a previous study conducted with Korean consumers visiting community pharmacies. Approximately, 69% of the consumers felt that DTCA for prescription drugs should be permitted under the condition of pre-approval of the DTCA contents by the Korean government or a third party [9]. The majority of students in this study, particularly the non-healthcare ones, also tended to display generally positive perceptions and attitudes towards DTCA for prescription drugs. As showed in previous studies, consumers with positive tendencies toward DTCA were likely to request advertised drugs more frequently [9,28–30]. Overall, it is expected that Korean consumers would ask their physicians for advertised drugs or would request them to prescribe these drugs if DTCA for prescription drugs is allowed in Korea. Consequently, in order to offer consumers drug advertisements with well-balanced and appropriate information, these should be systematically reviewed by the Korean government or independent third parties in order to prevent consumers from being exposed to advertisements with low credibility.

Additionally, the majority of students in both groups showed a tendency to agree with the idea that DTCA of prescription drugs should not be allowed on the Internet. Specifically, when compared to non-healthcare students (mean score, 3.12), healthcare students (mean score, 3.46) tended to agree more with this idea. This result is somewhat expected. Currently, the Internet is broadly used worldwide; therefore, electronic DTCA (eDTCA) through the Internet and social media platforms (e.g., Facebook, Twitter, and YouTube) is a rapidly growing marketing strategy that attracts the attention of pharmaceutical companies [13,31]. The potential benefits for pharmaceutical companies to use social media in their marketing strategies are various. In particular, they can quickly and economically reach diverse groups of future potential consumers with interactive and promotional activities that are difficult to utilize in traditional media channels, such as magazines, newspapers, and television. Additionally, consumers can also create promotional content, such as testimonials with their social media [13,31]. These are likely to be effective in attracting consumers’ attention that lead them to prefer specific drug brands. Consequently, false and misleading drug advertisements can be prevalent online, which may lead particular drugs to be prescribed to a large number of consumers, including those who are not at risk of the condition, and adverse drug events associated with those drugs are likely to occur. Policy makers should consider this point when possibly amending the current regulations that prohibit DTCA for prescription drugs in Korea.

Despite this study’s promising results, it must be mentioned that it has some limitations. The first limitation of this study was the representativeness of the survey participants. For example, most of the participants were current residents of Gwangju, located in the southwestern region of South Korea. Thus, it may be somewhat inappropriate to apply the findings of this study to the residents of other regions of South Korea. Secondly, the respondents’ non-demographic and lifestyle factors were not considered, but these could also affect perceptions and attitudes about DTCA for prescription drugs. Thirdly, DTCA for prescription drugs has not yet been legally allowed in Korea, but a large number of Koreans can actually obtain information on prescription drugs online through eDTCA. As a result, questions regarding eDTCA through the Internet and social media platforms should have been considered when the survey instrument was developed. Lastly, using prescription drugs usually increases with age; therefore, older people are likely to show different tendencies on perceptions and attitudes about DTCA for prescription drugs. In the well-designed future study, it is strongly encouraged to assess whether perceptions and attitudes about DTCA for prescription drugs differ between younger and older people.

## Conclusions

Although DTCA for prescription drugs is currently only approved in the U.S. and New Zealand, some promotional techniques are utilized in other countries, such as Korea. In this study, Korean college students’ perceptions and attitudes regarding DTCA for prescription drugs were investigated through a survey. The results of this study indicated that most students agreed that healthcare professionals had the responsibility to give information about prescription drugs to patients, and that DTCA for prescription drugs would not remove the rebates of pharmaceutical companies to doctors. The majority of students thought that DTCA for prescription drugs had to be permitted under the condition of pre-approval of the DTCA contents from the Korean government, and they also showed a tendency to think that prescription drugs should not be advertised on the Internet. Currently, while DTCA for prescription drugs is not allowed in Korea, a large number of Koreans can access information about prescription drugs through the Internet and social media platforms. Since DTCA for prescription drugs is a sensitive and controversial issue in Korea, it is recommended that the Korean government cautiously examine whether it should be permitted, after considering the current marketing strategies of pharmaceutical companies on the Internet and the effects of online eDTCA on Korean consumers.

## Supporting information

S1 File Questionnaire used in the survey. (PDF)

S2 File Complete set of data used in the analysis. (ZIP)
