# Supplementary material for: A survey of perceptions and attitudes about direct-to-consumer advertising of prescription drugs among college students in South Korea
Source: PLoS One. 2018 Jul 24;13(7):e0201108. doi: 10.1371/journal.pone.0201108 (PMC6057676; doi:10.1371/journal.pone.0201108)
Supplement: S1 File — (PDF) [file pone.0201108.s001.pdf]

Questionnaire

Hello? Thank you for your time. This questionnaire is intended to investigate perceptions and attitudes about direct-to-consumer advertising (DTCA) for prescription drugs among college students in South Korea. This questionnaire is anonymous, and the results will be used only for the purposes of research. We would really appreciate your sincere responses.

[Clinical Pharmacy Lab, Department of Pharmacy, Chosun University]

1. What is your gender? (   ) 1) Male 2) Female

2. How old are you? (   )

3. Which college do you belong in? (   )

1) Pharmacy 2) Medicine 3) Dentistry 4) Nursing 5) Natural sciences 6) Humanities

7) Law 8) Social sciences 9) Business 10) Engineering

11) Electronics and Information Engineering 12) Education 13) Health sciences 14) Foreign studies

15) Physical education 16) Art and design 17) General education

4. What is your academic year? (   )

1) Freshman 2) Sophomore 3) Junior 4) Senior

5. What is your current health status? (   )

1) Poor 2) Acceptable 3) Good 4) Very good

6. Have you heard about direct-to-consumer advertising (DTCA) for prescription drugs? (   )

1) Yes 2) No

Perceptions of college students about DTCA for prescription drugs (7-17)

5 = Strongly agree, 4 = Agree, 3 = Neutral, 2 = Disagree, 1 = Strongly disagree

No.	Questions	5	4	3	2	1
7	Do you think that doctors or pharmacists have to deliver information about prescription drugs to patients?					
8	Do you think that DTCA for prescription drugs can give patients confidence to talk with doctors about their concerns?					
9	Do you think that DTCA for prescription drugs can encourage patients to follow treatment instructions or advice from their doctors?					
10	Do you think that DTCA for prescription drugs can improve patients' awareness of medical conditions?					
11	Do you think that DTCA for prescription drugs can interfere with the relationships between patients and doctors?					
12	Do you think that DTCA for prescription drugs can promote unnecessary visits to hospitals?					
13	Do you think that DTCA for prescription drugs can prevent incorrect information on drugs from being spread?					

14	Do you think that DTCA for prescription drugs can broaden patients' choices for using drugs?					
15	Do you expect that DTCA for prescription drugs will increase the profits of pharmaceutical companies?					
16	Do you think that DTCA for prescription drugs can play a role in removing the rebates of pharmaceutical companies to doctors?					
17	Do you think that DTCA for prescription drugs can have a negative effect on patients' drug misuse/abuse?					

Attitudes of college students toward DTCA for prescription drugs (18-27)

5 = Strongly agree, 4 = Agree, 3 = Neutral, 2 = Disagree, 1 = Strongly disagree

No.	Questions	5	4	3	2	1
18	Do you think that DTCA for prescription drugs is necessary for patients?					
19	Do you think that information about drugs provided by DTCA is reliable?					
20	Are you willing to actively recommend DTCA for prescription drugs when patients ask you about it in the future?					
21	Are you willing to actively utilize the data obtained from DTCA for prescription drugs when consulting patients in the future?					
22	Are you willing to actively accept patients' opinions when they ask you to prescribe, fill, or administer drugs which they have seen on the DTCA in the future?					
23	Do you think that DTCA for prescription drugs should not be permitted on the Internet?					
24	Do you think that DTCA for prescription drugs can create unrealistic expectations about drugs?					
25	Do you think that DTCA for prescription drugs can improve patients' drug compliance?					
26	Do you expect that DTCA for prescription drugs will lead to lowering drug prices due to increased market competition?					
27	Do you think that the government should mandate pre-approval of all DTCAs for prescription drugs if they are permitted?					

Thank you very much for your sincere participation in the survey.

설 문 지

안녕하세요? 귀하의 소중한 시간을 내어 주셔서 감사합니다. 본 설문지는 국내 대학생들의 전문의약품의 직접적인 소비자 광고(direct-to-consumer advertising, DTCA)에 대한 인식 및 태도를 조사하기 위한 설문입니다. 본 설문지는 무기명으로 작성되며, 연구 이외의 목적에는 사용되지 않음을 약속드립니다. 수고스럽지만, 성의껏 답해 주시면 감사하겠습니다. [조선대학교 약학대학 임상약학실]

1. 귀하의 성별은? (　　) 1) 남성 2) 여성

2. 귀하의 나이는? (만　　세)

3. 현재 귀하의 소속대학은? (　　)
1) 약학대학 2) 의과대학 3) 치과대학 4) 간호대학 5) 자연과학대학 6) 인문과학대학
7) 법과대학 8) 사회과학대학 9) 경상대학 10) 공과대학 11) 전자정보공과대학 12) 사범대학
13) 보건과학대학 14) 외국어대학 15) 체육대학 16) 미술대학 17) 기초교육대학

4. 현재 소속대학에서 귀하의 학년은? (　　)
1) 1학년 2) 2학년 3) 3학년 4) 4학년

5. 현재 귀하의 건강상태는? (　　)
1) 안 좋다 2) 보통이다 3) 좋다 4) 매우 좋다

6. 전문의약품에 대한 직접광고(DTCA)를 들어본 적이 있습니까?
1) 예　　2) 아니오

<u>대학생들의 전문의약품 DTCA에 대한 인식(문항 7-17)</u>

5 = 매우 그렇다 4 = 그런 편이다 3 = 보통이다 2 =그렇지 않은 편이다 1 = 매우 그렇지 않다

번 호	평가항목	5	4	3	2	1
7	귀하는 전문의약품 정보는 의사나 약사가 소비자에게 전달해야 한다고 생각하십니까?					
8	귀하는 소비자들이 DTCA를 통해 의사와 자신의 상태에 대하여 상담을 할 경우에 자신감을 얻을 수 있다고 생각하십니까?					
9	귀하는 DTCA가 소비자들에게 의사들의 치료지침과 충고를 잘 따르도록 할 수 있다고 생각하십니까?					
10	귀하는 DTCA가 소비자들의 질병에 대한 인식을 향상시킬 수 있다고 생각하십니까?					
11	귀하는 DTCA가 소비자와 의사들의 관계를 방해할 수 있다고 생각하십니까?					
12	귀하는 DTCA가 필요치 않은 병원방문의 횟수를 증가시킬 수 있다고 생각하십니까?					
13	귀하는 DTCA가 약물에 대한 잘못된 정보가 확산되는 것을 방지할 수 있다고 생각하십니까?					
14	귀하는 DTCA가 약물 사용에 대한 소비자들의 선택의 폭을 넓힐 수 있다고 생각하십니까?					
15	귀하는 DTCA가 제약회사의 이윤을 증대시킬 것이라고 기대하십니까?					
16	귀하는 DTCA가 의사에 대한 제약회사의					

	리베이트를 제거할 수 있는 역할을 할 수 있다고 생각하십니까?					
17	귀하는 DTCA가 소비자들의 약물에 대한 오남용에 부정적인 영향을 미칠 수 있다고 생각하십니까?					

<u>대학생들의 전문의약품 DTCA에 대한 태도(문항 18-27)</u>

5 = 매우 그렇다 4 = 그런 편이다 3 = 보통이다 2 = 그렇지 않은 편이다 1 = 매우 그렇지 않다

번 호	평가항목	5	4	3	2	1
18	귀하는 DTCA가 소비자들에게 필요하다고 생각하십니까?					
19	귀하는 DTCA에서 제공하는 의약품에 대한 정보가 신뢰성이 있다고 생각하십니까?					
20	귀하는 미래에 소비자들이 DTCA에 대해 질문할 경우 적극적으로 추천할 의향이 있습니까?					
21	귀하는 미래에 소비자들을 상담할 경우 DTCA에서 얻은 의약품에 대한 자료를 적극적으로 활용할 의향이 있습니까?					
22	귀하는 미래에 소비자들이 DTCA에서 본 의약품의 처방이나 조제 또는 투약을 요구할 경우 그들의 의견을 적극적으로 수용할 의향이 있습니까?					
23	귀하는 인터넷상에 DTCA를 허용하지 말아야 한다고 생각하십니까?					
24	귀하는 DTCA가 소비자들에게 비현실적인 기대감을 줄 수 있다고 생각하십니까?					
25	귀하는 DTCA가 소비자들의 약물 순응도를 향상시킬 수 있다고 생각하십니까?					
26	귀하는 DTCA가 시장 경쟁의 증가로 인해 의약품 가격을 낮출 것이라고 기대하십니까?					
27	만약, 정부가 모든 처방조제 의약품에 대한 DTCA를 허용한다면, 귀하는 정부가 의무적으로 DTCA에 대한 사전심의를 해야 한다고 생각하십니까?					

<u>성의껏 질문에 참여해 주셔서 대단히 감사합니다.</u>
